# Nanoparticle Dynamics in Composite Hydrogels Exposed to Low-Frequency Focused Ultrasound

**DOI:** 10.3390/gels9100771

**Published:** 2023-09-22

**Authors:** Caroline Einen, Sebastian E. N. Price, Kim Ulvik, Magnus Aa. Gjennestad, Rune Hansen, Signe Kjelstrup, Catharina de Lange Davies

**Affiliations:** 1 Porelab and Department of Physics, The Norwegian University of Science and Technology (NTNU), 7491 Trondheim, Norway; 2 Porelab and Department of Chemistry, The Norwegian University of Science and Technology (NTNU), 7491 Trondheim, Norway; 3 Department of Physics, The Norwegian University of Science and Technology (NTNU), 7491 Trondheim, Norway; 4 Porelab and SINTEF Energy Research, 7034 Trondheim, Norway; 5 Department of Health Research at SINTEF, 7465 Trondheim, Norway; 6 Department of Circulation and Medical Imaging, The Norwegian University of Science and Technology (NTNU), 7491 Trondheim, Norway

**Keywords:** hydrogel, focused ultrasound (FUS), single-particle tracking (SPT), acoustic radiation force (ARF), extracellular matrix (ECM) model, nanoparticles, drug delivery

## Abstract

Pulsed focused ultrasound (FUS) in combination with microbubbles has been shown to improve delivery and penetration of nanoparticles in tumors. To understand the mechanisms behind this treatment, it is important to evaluate the contribution of FUS without microbubbles on increased nanoparticle penetration and transport in the tumor extracellular matrix (ECM). A composite agarose hydrogel was made to model the porous structure, the acoustic attenuation and the hydraulic conductivity of the tumor ECM. Single-particle tracking was used as a novel method to monitor nanoparticle dynamics in the hydrogel during FUS exposure. FUS exposure at 1 MHz and 1 MPa was performed to detect any increase in nanoparticle diffusion or particle streaming at acoustic parameters relevant for FUS in combination with microbubbles. Results were compared to a model of acoustic streaming. The nanoparticles displayed anomalous diffusion in the hydrogel, and FUS with a duty cycle of 20% increased the nanoparticle diffusion coefficient by 23%. No increase in diffusion was found for lower duty cycles. FUS displaced the hydrogel itself at duty cycles above 10%; however, acoustic streaming was found to be negligible. In conclusion, pulsed FUS alone cannot explain the enhanced penetration of nanoparticles seen when using FUS and microbubbles for nanoparticle delivery, but it could be used as a tool to enhance diffusion of particles in the tumor ECM.

## 1. Introduction

Encapsulation of therapeutics in nanoparticles for cancer therapy is a promising strategy for increased drug delivery and reduced toxic effects in normal tissues [1,2,3]. This is partly motivated by the enhanced permeability and retention (EPR) effect, in which increased permeability of tumor vessels and a malfunctioning lymphatic system could lead to passive accumulation and retention of macromolecules in tumors [4]. However, recent studies have found that only a low fraction of the injected nanoparticle dose ends up in the tumor [5] and that trans-endothelial pathways as opposed to particles escaping through blood vessel gaps might be the main mechanism of particle extravasation [6]. Following extravasation, the nanoparticles must penetrate and distribute into the extracellular matrix (ECM) and dense tumor stroma, where transport is mostly limited to diffusion due to a high interstitial fluid pressure [7,8]. Thus, nanoparticles are heterogeneously distributed in tumor tissue and are mainly close to the capillary wall [9].

Focused ultrasound (FUS) and microbubbles (MBs) have emerged as a promising strategy to increase the uptake and penetration of nanoparticles in tumors, where the local cavitation of MBs has been shown to enhance the permeability and penetration of co-injected [10,11,12] or co-formulated [13] nanoparticles in target tissues. Emphasis is often placed on cavitation as the main contributor to nanoparticle delivery [14,15]. However, the acoustic radiation force (ARF) could play a role in improving the penetration of nanoparticles through the ECM, and its contribution to nanoparticle delivery alongside cavitation must be understood for optimal tuning of the acoustic parameters. The ARF arises from momentum transfer from the ultrasound wave to the tissue due to scattering and absorption. The force increases with increasing ultrasound intensity and frequency and material acoustic attenuation. The ARF can displace particles and tissues [16,17], but it can also induce acoustic streaming of fluids which could enhance nanoparticle transport in the ECM [18,19]. With nanosized particles and ultrasound frequencies around 1 MHz, the ARF on the particle itself would be small, and the main driving force behind particle transport would likely be from acoustic streaming of the fluid [20,21].

The effect of FUS and the ARF on the penetration of nanoparticles has been demonstrated in several studies in vivo. Frenkel et al. [22] observed an increase in the penetration of nanoparticles in fish skin. By confocal imaging of nanoparticles in tumor sections, our group [23] found that the ARF improved the penetration of nanoparticles into the ECM from the nearest blood vessel. The ARF has also been studied in vitro using hydrogels. El Ghamrawy et al. [19,24] investigated acoustic streaming in a macroporous polyacrylamide (MPPa) hydrogel by measuring dye clearance using a video camera. Lea-Banks et al. [20] investigated how FUS in the presence or absence of MBs could push nanoparticles of different densities from a water phase into an agarose hydrogel. Our group [21] used confocal laser scanning microscopy to examine the displacement of nanoparticles in collagen gels under the influence of the ARF. Interestingly, we found no effect on nanoparticle displacement but rather a deformation of the gel itself. In vitro studies on the effect of FUS on the diffusion of nanoparticles have also been performed. Ma et al. [25] and Karki et al. [26] measured nanoparticle diffusion coefficients in an agarose hydrogel by analyzing particle concentration distributions through fluorescent imaging and estimated an additional contribution to diffusion from ultrasound exposure. 

In this work, the effect of pulsed FUS on nanoparticle diffusion and acoustic streaming was studied in composite agarose hydrogels mimicking the porous structure of the ECM, and the attenuating properties and the hydraulic conductivity of the gel were measured. Although the ARF increases with increasing frequency, we applied pulsed FUS at 1 MHz, a frequency commonly used to oscillate MBs in FUS- and MB-mediated nanoparticle delivery. This was to evaluate whether pulsed FUS alone at this low frequency could contribute to the increased penetration of nanoparticles in the ECM.

To study the movement of nanoparticles during FUS exposure, we used single-particle tracking (SPT). SPT is a useful method to investigate dynamics of small particles, in which particles are imaged over time using light or fluorescence microscopy. Different algorithms can be used to both localize individual particle centroids and connect these between frames to form particle trajectories [27]. The mean squared displacement (MSD) of particles can then be analyzed to directly obtain information on both particle diffusion and drift velocity [28,29,30]. SPT has been used in a wide variety of applications including the investigation of particle diffusion and dynamics in hydrogels and biofilms as well as the movement of molecules in cell membranes [31,32,33,34]. However, SPT has to our knowledge not been used to study nanoparticle movement in hydrogels during FUS exposure. By using SPT and comparing our experimental results with our recently developed model of acoustic streaming of fluid in a porous medium [35], we found that low-frequency pulsed FUS above a certain duty cycle (DC), the time percentage the ultrasound is on, could increase the particle diffusion but not induce any notable acoustic streaming. 

## 2. Results

### 2.1. Hydrogel Characterization

#### 2.1.1. Increasing Acoustic Attenuation of Agarose Hydrogels with Additives

The ARF is proportional to the acoustic attenuation by the material, and the attenuation of agarose hydrogels can be increased through incorporation of attenuating components in the gel. Evaporated milk (10% (*v*/*v*) and 20 % (*v*/*v*)) or bovine serum albumin (BSA) (10% (*w*/*v*) and 20% (*w*/*v*)) was added to the hydrogel and the increase in acoustic attenuation was measured by an insertion technique. The additives increased the acoustic attenuation of the agarose hydrogels (Table 1), corresponding with previous studies [36,37]. Increasing the concentration from 2% (*w*/*v*) agarose to 5% (*w*/*v*) did not have a large impact on the acoustic attenuation at 1 MHz, but it did increase the attenuation at higher frequencies. The measured values were slightly lower than what would be expected for soft tissues, averaging at around 0.5 dB/cm at 1 MHz [38]. However, to avoid an agarose gel that is too dense and to maintain an adequate optical transparency, a 2% (*w*/*v*) agarose gel with 10% (*v*/*v*) evaporated milk with an acoustic attenuation of 0.14 ± 0.02 dB/cm at 1 MHz was used as a compromise for all further experiments, referred to as the agarose–milk hydrogel. 

#### 2.1.2. Distribution of Evaporated Milk and Hydrogel Water Content

**Figure 1:**
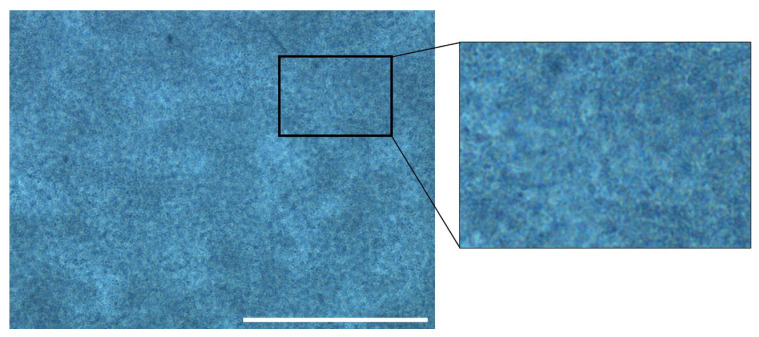
Imaging agarose–milk hydrogel. Representative phase contrast image of an agarose–milk hydrogel showing the dispersion of milk globules in the gel taken with a 40X lens. The white scale bar is 100 μm and the cut-out box shows a zoomed in area.

The distribution of evaporated milk globules in the agarose–milk hydrogel was imaged by phase contrast light microscopy (Figure 1). The images showed a heterogeneous distribution of darker spots with diameters between 7 and 17 μm (*n* = 30). The agarose–milk hydrogel had a water mass fraction of 0.953 ± 0.002 (*n* = 6), which was estimated by comparing the dry weight and wet weight of the hydrogel. 

#### 2.1.3. Hydraulic Conductivity of Agarose–Milk Hydrogels

**Figure 2:**
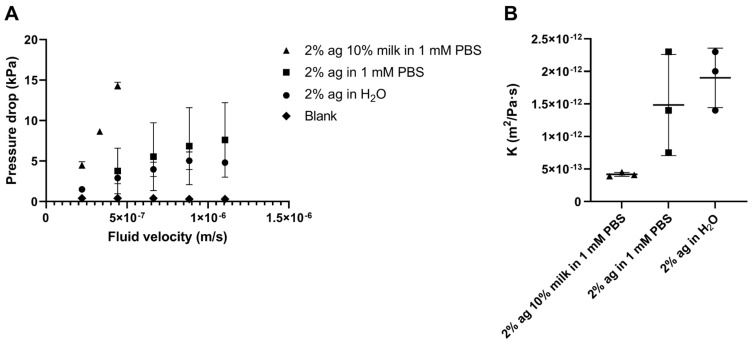
Hydraulic conductivity. Mean steady-state pressure drop (**A**) plotted against fluid velocity through the sample for agarose gels with evaporated milk in phosphate buffered saline (PBS) (2% ag 10% milk in 1 mM PBS) as well as gels comprising agarose only in PBS (2% ag in 1 mM PBS) and water (2% ag in H_2_O). The measured pressure drop across a sample tube filled with 1 mM PBS (blank) is included to confirm low flow resistance through the setup itself. Data points indicate mean values and error bars show standard deviations. Hydraulic conductivity (K) values (**B**) were estimated by the Darcy equation (Equation (3)). Horizontal lines indicate mean values, error bars show standard deviation and data points show individual samples (*n* = 3).

The hydraulic conductivity of agarose and agarose–milk hydrogels was measured by monitoring the steady-state pressure drop of liquid flowing at a constant rate through the gel (Figure 2). Replacing 10% of the liquid volume in the agarose hydrogel with evaporated milk gave roughly a threefold reduction in the hydraulic conductivity compared to hydrogels composed of agarose only in 1 mM phosphate buffered saline (PBS), from (1.5 ± 0.7)⋅10^−12^ to (0.41 ± 0.09)⋅10^−12^ m^2^/Pa⋅s. Agarose made with water had a similar but slightly higher hydraulic conductivity compared to agarose in 1 mM PBS, with conductivity at (1.9 ± 0.5)⋅10^−12^ m^2^/Pa⋅s. Measured values for agarose only were in order of magnitude comparable to other studies [39,40,41]. The hydraulic conductivity of the agarose–milk hydrogel also fell within the range of the conductivity of tumor tissues, where values have been measured between 10^−14^ and 10^−12^ m^2^/ Pa⋅s [42,43,44]. The similar hydraulic conductivity between the agarose–milk hydrogel and tissue indicate that the gel is a suitable model for evaluation of acoustic streaming in tumors. 

### 2.2. Effect of Pulsed FUS on Nanoparticle Movement

#### 2.2.1. Nanoparticle Diffusion in Buffer and in Agarose–Milk Hydrogels

**Figure 3:**
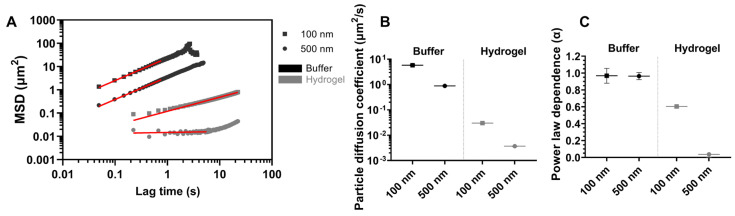
Diffusion of nanoparticles. (**A**) Log–log plot of ensemble mean squared displacement (MSD) of 100 nm and 500 nm polystyrene nanoparticles freely moving in buffer of 1 mM PBS (black symbols) or in the agarose–milk hydrogel (grey symbols) estimated from single-particle tracking (SPT), where the solid red lines show the fitted diffusion model (Equation (4)). The fitted diffusion coefficients (**B**) and power law coefficients α (**C**), comparing the nanoparticles in buffer and in the hydrogel. Data points (**B**,**C**) indicate fitted values with error bars being the 95% confidence interval.

SPT was used to estimate diffusion coefficients of 100 nm and 500 nm particles in 1 mM PBS and in the agarose–milk hydrogel by fitting a diffusion model (Equation (4)) to the ensemble particle MSD (Figure 3). In buffer, both the 100 nm and 500 nm particles displayed pure Brownian motion by an approximately linear dependence between time and ensemble MSD, with fitted power law coefficients α of 0.97 and 0.96, respectively. Resulting ensemble diffusion coefficients were estimated as 5.8 μm^2^/s and 0.9 μm^2^/s for the 100 nm and 500 nm particles, respectively. When the 100 nm nanoparticles were embedded in the agarose–milk hydrogel, they displayed anomalous diffusion with an ensemble α of 0.60 and an over 100-fold lower ensemble diffusion coefficient of 0.030 μm^2^/s compared to the freely diffusing particles. For the 500 nm particles in the hydrogel, the diffusion model could only be fitted to lag times up to 5.6 s due to increasing levels of noise. Here, curve fitting gave an α of 0.04 and a diffusion coefficient of 0.004 μm^2^/s, rendering the 500 nm particles effectively immobilized in the agarose–milk hydrogel.

#### 2.2.2. Pulsed FUS Increased Nanoparticle Diffusion

**Figure 4:**
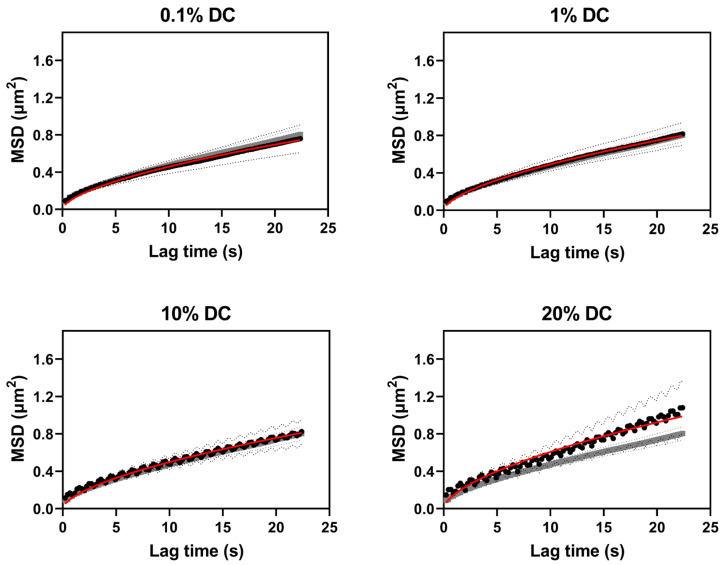
Ensemble MSD of nanoparticles in agarose–milk hydrogels during pulsed focused ultrasound exposure. Ensemble MSD of 100 nm nanoparticles (black circles) in agarose–milk hydrogels exposed to focused ultrasound (FUS) with 0.1 to 20% duty cycle (DC), with the fitted diffusion model (red line) (Equation (4)). Grey curves show ensemble MSD of nanoparticles in agarose–milk hydrogels not exposed to FUS for reference. Dotted lines indicate 95% confidence interval.

**Figure 5:**
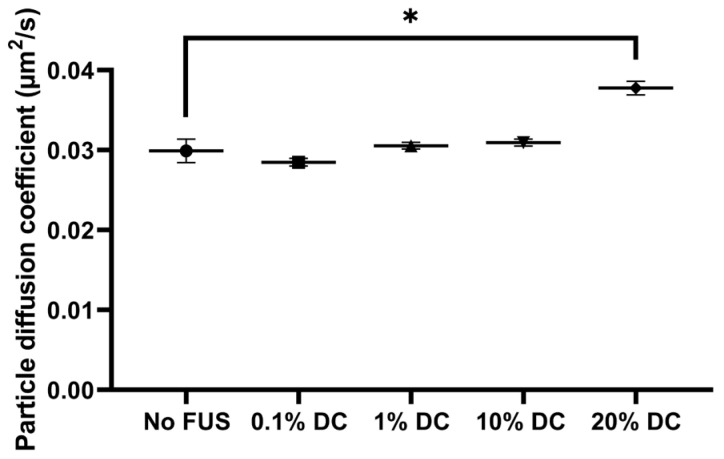
Nanoparticle diffusion coefficients with and without pulsed FUS. Ensemble nanoparticle diffusion coefficients estimated from curve fitting to ensemble MSD curves (Figure 4) for 100 nm nanoparticles in agarose–milk hydrogels exposed to no FUS or to FUS with a DC from 0.1 to 20%. Data points show fitted values and error bars show the 95% confidence interval in the fit. Asterisks (*) indicate a statistically significant increase compared to the no FUS value (*p* = 0.05).

SPT was used to estimate diffusion coefficients of the 100 nm nanoparticles in the agarose–milk hydrogel during pulsed FUS exposure (Figure 4). FUS with 0.1% and 1% DCs did not visibly change the ensemble MSD compared to the MSD of nanoparticles not exposed to FUS. The 10% and 20% DC MSD curves were more susceptible to noise. Furthermore, especially the 20% DC curve separated from the no FUS case. The fitted diffusion coefficients of particles in agarose–milk hydrogels exposed to FUS were similar to the no FUS diffusion coefficient of 0.030 μm^2^/s for all DCs except the 20% DC (Figure 5). The ensemble nanoparticle diffusion coefficients were 0.028 μm^2^/s, 0.030 μm^2^/s and 0.031 μm^2^/s with 0.1%, 1% and 10% DCs, respectively. Applying FUS with a 20% DC gave a significant increase in particle diffusion by 23%, with an ensemble diffusion coefficient of 0.037 μm^2^/s. No increase in temperature in the buffer solution surrounding the hydrogel when applying FUS with 0.1% or 1% DCs was detected. Conversely, the solution temperature rose on average by 0.6 ± 0.3 °C and 0.9 ± 0.3 °C with 10% and 20% DCs, respectively.

#### 2.2.3. Acoustic Streaming vs. Hydrogel Displacement

**Figure 6:**
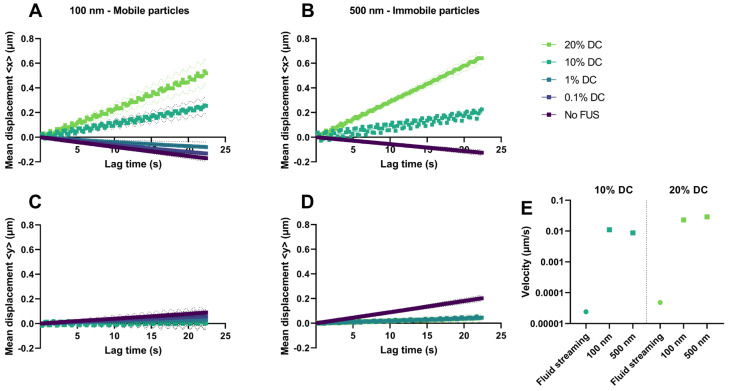
FUS-induced displacement of hydrogels. Mean displacement (MD) of nanoparticles in the x direction parallel to the ultrasound field for the mobile 100 nm particles (**A**) and the immobile 500 nm particles (**B**) in the agarose–milk hydrogel with FUS exposure of varying DCs. MD in the y direction orthogonal to the ultrasound field for the 100 nm (**C**) and the 500 nm (**D**) particles. Dotted lines (**A**–**D**) indicate 95% confidence intervals. (**E**) Simulated acoustic streaming of the fluid in the hydrogel (fluid streaming) (Equation (9)) for the 10% and 20% DCs compared to the displacement velocities of the 100 nm and 500 nm nanoparticles.

The ARF can induce an acoustic streaming of the fluid inside the agarose–milk hydrogel, but it can also induce a displacement of the hydrogel itself. To separate the two effects, the ensemble nanoparticle mean displacement (MD) during FUS exposure was estimated and compared for both mobile 100 nm and immobilized 500 nm particles in the ultrasound propagation direction (positive x direction) (Figure 6). Samples not exposed to FUS showed a slightly negative particle drift in the x direction (Figure 6A,B) and a positive drift in the y direction for both particle sizes (Figure 6C,D), indicating sources of drift in the experimental setup itself. Similar behavior was found for the 100 nm nanoparticles in the hydrogels exposed to FUS with 0.1% and 1% DC. Application of FUS with 10% and 20% DCs gave an MD of nanoparticles in the ultrasound propagation direction for both the 100 nm and the 500 nm particles, indicating that FUS displaced the hydrogel. The slope of the MD curve in the x direction was used to estimate particle displacement velocities (Figure 6E), giving velocities of 0.011 μm/s and 0.009 μm/s with a 10% DC and 0.023 μm/s and 0.029 μm/s with a 20% DC for the 100 nm and 500 nm particles, respectively. 

To obtain information about potential acoustic streaming in the hydrogel, simulations were performed. Estimated streaming velocities (Equation (9)) were 1000-fold lower in magnitude compared to the measured nanoparticle displacement velocities in the hydrogels exposed to FUS with 10% or 20% DCs (Figure 6E). Any contribution from the acoustic streaming of the buffer above the gel that would increase the flow velocity in the buffer–gel interphase was also estimated and found to be negligible at the depth of imaging (see Appendix A). 

## 3. Discussion

The motivation behind using a composite agarose hydrogel was to create an in vitro model of the tumor ECM, with a similar acoustic attenuation and hydraulic conductivity as tissue, to evaluate any FUS-induced increase in nanoparticle diffusion or a potential acoustic streaming of fluid with particles in the gel. While the acoustic attenuation of the agarose–milk hydrogel was slightly lower than the average for soft tissues, a hydraulic conductivity comparable to tumors was achieved. Further, addition of evaporated milk to the agarose gels for increased attenuation lowered the hydraulic conductivity, thereby giving a trade-off in optimization of the two parameters. The acoustic streaming velocity in a porous medium is approximately linearly related to both the hydraulic conductivity and the acoustic attenuation [35], so an increase in one of the parameters is only useful if the other does not decrease correspondingly. In any case, it is important to be aware of the limitations within the model used to mimic the tumor ECM. While the agarose–milk hydrogel models the acoustic attenuation and hydraulic conductivity of tissue, it will not adequately represent the dynamic, heterogenous and chaotic nature of the tumor ECM.

SPT was chosen in our study to monitor nanoparticle dynamics as it provided direct insight into particle diffusion and displacement during FUS exposure. Other techniques such as diffusion nuclear magnetic resonance (NMR) spectroscopy [45] or diffusion-weighted magnetic resonance imaging (MRI) [46] have been used to characterize particles and measure their diffusion coefficient or to measure diffusion of magnetic particles in various tissues to monitor therapeutic effects, respectively. However, these techniques cannot be applied during FUS exposure, and can therefore only measure the permanent or long-lasting effects of FUS. Fluorescence correlation spectroscopy (FCS) [47] can be applied during FUS exposure to determine particle diffusion coefficients but cannot easily separate and determine the particle drift velocity such as in SPT. One could also monitor concentration profiles of nanoparticles in the gel before and after FUS exposure [21,25,26], but this does not give the in situ knowledge SPT provides. SPT is, however, vulnerable to noise such as vibrations in the setup, which limits the ultrasound intensity that can be investigated with this technique. Noise became increasingly present when using DCs of 10% and 20%, and using higher DCs was deemed infeasible. 

Diffusion of nanoparticles through the ECM is limited by steric hindrance, geometric tortuosity and electrostatic interactions, here provided by the agarose fibers, fat globules and milk proteins in the agarose–milk hydrogel. Compared to diffusion in a free buffer, 100 nm particles in the hydrogel displayed anomalous diffusion with a subdiffusive nature and a 100-fold reduction in the diffusion coefficient. The larger 500 nm particles were effectively immobilized. This could be attributed to the size of the nanoparticles relative to the mesh size of the agarose–milk hydrogel. Diffusion coefficients of macromolecules and nanoparticles in 1.5–2% (*w*/*v*) agarose gels have been shown to drop dramatically at a hydrodynamic radius above 20–30 nm [48,49]. Pluen et al. [48] measured the diffusion coefficient of polymer particles 103 nm in diameter to be roughly 100 times lower in 2% agarose gels compared to free solution, in good agreement with the results from our study. Furthermore, the pore sizes of 2% (*w*/*v*) agarose gels are reported to be in ranges from 100 to 600 nm [50,51]. While the influence on pore size of replacing 10% of the liquid volume with evaporated milk has not been evaluated, it would be expected for the large 500 nm particles to be immobile in the agarose–milk hydrogel. 

Pulsed FUS increased the ensemble nanoparticle diffusion coefficient by 23% at a 20% DC. No increase in diffusion was observed at lower DCs, indicating a threshold. Diffusion is known to increase with absolute temperature, and the increase in the nanoparticle diffusion coefficient could be partly due to local heating of the hydrogel. As the hydrogel absorbs the ultrasound, most of the deposited energy will dissipate as heat in the gel, where the amount of heat deposited is proportional to the intensity of the ultrasound. When using pulsed FUS, however, heat will dissipate while the ultrasound is off. Thus, there will be a threshold for net temperature increase at the focal area depending on the DC for a given intensity and pulse repetition frequency (PRF). Assuming the Stokes–Einstein equation for diffusion of nanoparticles in water, which takes into consideration the absolute temperature and a temperature-dependent viscosity, a 23% increase in the diffusion coefficient requires an increase in temperature of approximately 10 °C. It is challenging to measure the temperature in the focal spot, both due to the accuracy needed in positioning and the interference a thermocouple or other insertion instrument would have with the ultrasound field [52]. However, an average temperature increase in the buffer solution of 0.6 °C and 0.9 °C was detected after a 5 min FUS treatment with 10% and 20% DCs, respectively, indicating that some heating of the agarose–milk hydrogel likely occurred. 

Another source for the increase in nanoparticle diffusion could be from a phenomenon called oscillatory diffusion [53]. The oscillation of a particle, for example by an acoustic field, that experiences random hindering could on average lead to increased particle movement as the oscillation can un-trap hindered particles [54,55]. A model of oscillatory diffusion was used by Karki et al. [26] to experimentally determine FUS-enhanced diffusion coefficients of nanoparticles in agarose gels, based on measured nanoparticle concentration profiles. They found that the effective diffusion coefficient could increase by nearly an order of magnitude for small 20 nm or 40 nm particles when applying pulsed low-pressure FUS (up to 0.18 MPa peak negative pressure, 0.5–2.5 MHz frequency, 10% DC). The 100 nm particles used in our study clearly experienced random hindering in the agarose–milk hydrogel, and oscillatory diffusion could have contributed to the increased diffusion coefficient measured with FUS using 20% DC. 

Another factor to consider is whether pulsed FUS could change the structure of the agarose–milk hydrogel itself. Such changes might provide more space for the nanoparticles to diffuse and could also increase acoustic streaming, although it was not possible to observe these changes in our study. Similarly, FUS applied in vivo might change the structure of the tumor ECM by breaking or modifying linker proteins, the collagen network or the gel of glycosaminoglycans. However, changes in the tumor microenvironment have not been reported for low-intensity FUS, but a few studies using high ultrasound intensities report changes in the collagen network and reduced interstitial fluid pressure [56,57]. 

FUS is reported to induce acoustic streaming and tissue displacement. A net displacement velocity between 0.01 and 0.02 μm/s of 100 nm nanoparticles during FUS exposure at 10 and 20% DCs was found, but it was not necessarily a measure of acoustic streaming in the agarose–milk hydrogel. Using our previously validated model of acoustic streaming in a porous medium [35], the fluid velocity in the hydrogel was predicted to be as low as approximately 0.05 ⋅10^−3^ μm/s. We also estimated that any contribution to the measured displacement velocity from streaming in the buffer above the hydrogel was negligible. Furthermore, the displacement velocity of immobile 500 nm particles in the hydrogel during FUS exposure was the same as that of the mobile 100 nm particles. Therefore, it is likely that the measured particle displacement velocities reflect a small displacement of the hydrogel itself due to the ARF. Deformation of materials due to the ARF is known, and the degree of deformation when subjected to a given force is dependent on the viscoelastic properties of the material [17]. Our group observed this previously when applying pulsed FUS with a higher frequency (10 MHz) to soft collagen hydrogels (elastic modulus of 0.24 kPa), where the FUS created a local indentation in the hydrogel surface [21]. However, in the present study using much stiffer agarose gels, with the elastic modulus for 2% (*w*/*v*) agarose measured as 235 kPa [58], we measured a very small hydrogel displacement not visible upon inspection of the gel.

Others have been able to measure acoustic streaming in hydrogels, namely El Ghamrawy et al. [19]. Here, the authors investigated streaming of a small molecular dye in MPPa hydrogels, where they measured a significant streaming of the dye out of the hydrogel in the order of 1–100 μm/s. There are a few key differences between the El Ghamrawy et al. study and our study. Firstly, the hydrogel composition is different, where the MPPa gels have much larger pore sizes and permeability compared to the agarose–milk gel. The acoustic intensities employed were also 100–1000 times larger compared to those used in this study. While El Ghamrawy et al. demonstrate that acoustic streaming in a porous medium is feasible, the results in our study show that streaming is unlikely to happen when using low-intensity pulsed FUS in a material with a similar hydraulic conductivity as tumor tissue.

## 4. Conclusions

The results from this study indicate that pulsed low-frequency FUS can increase nanoparticle diffusion when using a high DC but not induce any acoustic streaming at ultrasound parameters relevant for FUS- and MB-mediated delivery of nanoparticles. When using FUS and MBs for drug delivery, it is common practice to use a low DC and a low PRF to allow for reperfusion of MBs into the tumor volume. It is unlikely that FUS without MBs can enhance diffusion or induce streaming of nanoparticles that fully explain the improved particle penetration observed when using FUS and MBs. However, given the results from this study, one could consider applying a second FUS exposure with a higher DC or frequency after the FUS and MB treatment to enhance diffusion of particles extravasated into the tumor ECM. 

## 5. Materials and Methods

### 5.1. Nanoparticles

Fluorescent polystyrene nanoparticles with a carboxylated surface and diameters of 100 nm and 500 nm (FluoSpheres^®^ carboxylate-modified microspheres, red fluorescent (580/605), Thermo Fisher Scientific, Waltham, MA, USA) were used. Dynamic light scattering measurements (Zetasizer ZS, Malvern Panalytical Ltd., Malvern, UK) revealed that the nanoparticles had a narrow size distribution with a Z-average diameter of 116.9 ± 0.9 nm and 465 ± 2 nm with polydispersity indexes of 0.02 ± 0.01 and 0.01 ± 0.01 for the 100 nm and 500 nm particles, respectively (*n* = 3). In 1 mM PBS, the particle zeta-potentials were −51 ± 1 mV and −85 ± 2 mV, respectively (*n* = 3).

### 5.2. Preparation of Composite Agarose Hydrogels

Agarose powder (Agarose, BioReagent, for molecular biology, low EEO, Sigma-Aldrich, St. Louis, MO, USA) was added to deionized (DI) water or a weak buffer of 1 mM PBS (tablets, Sigma-Aldrich) at pH 7.4, to achieve an end agarose concentration of 2% (*w*/*v*) or 5 % (*w*/*v*). The solution was heated during stirring to dissolve the agarose and subsequently placed in a 50–55 °C water bath for 20 min to cool. DI water or buffer at 50 °C was added to account for solvent evaporation if needed. To increase acoustic attenuation, either evaporated milk (Tørsleffs^®^ condensed milk, unsweetened, Hvidovre, Denmark) or BSA (Sigma-Aldrich) was heated to 50°C and added at an appropriate concentration. The evaporated milk was filtered for small particles (Grade 4 Whatman, Sigma-Aldrich) before use and used within 3 days post-opening. BSA was first dissolved in DI water prior to addition to the agarose solution. For gels used in nanoparticle diffusion and FUS experiments, polystyrene nanoparticles were added in this step to a concentration of 5 μg/mL (100 nm) or 400 μg/mL (500 nm). The agarose solution was subsequently stirred for 10 min prior to degassing in a desiccator for 8–10 min. The solution was cast in an appropriate mold depending on the experiment and left at room temperature for 5–10 min to solidify before adding DI water or 1 mM PBS to prevent drying of the gel. All samples containing evaporated milk were used the day of preparation. Samples with agarose only or with BSA were kept refrigerated at 4 °C for a maximum of 4 days and tempered to room temperature before use.

### 5.3. Hydrogel Characterization

#### 5.3.1. Acoustic Attenuation Measurements

**Figure 7:**
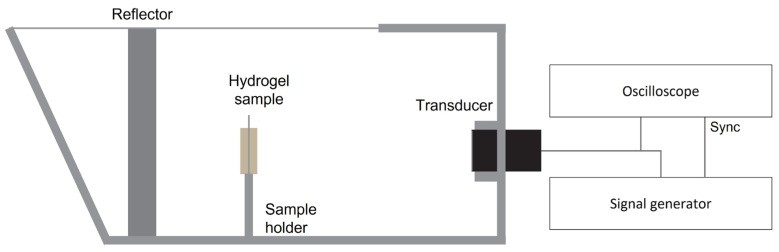
Measurement of acoustic attenuation. Schematic of setup for measuring acoustic attenuation, where the hydrogel sample is placed in a custom water tank. A signal generator drives a transducer, sending a short pulse through the hydrogel sample that is reflected by a thick acrylic slab back through the sample to be received by the transducer. The received pulse was logged by an oscilloscope and compared to a reference measurement.

Evaporated milk (10% (*v*/*v*) and 20% (*v*/*v*)) or BSA (10% (*w*/*v*) and 20% (*w*/*v*)) was added to the hydrogel and the increase in acoustic attenuation was measured by an insertion technique. Agarose solutions were cast into custom U-shaped acrylic molds 25 mm in width, 30 mm in height and 5 mm thick. The front and back walls of the mold were covered with thin plastic film to ensure acoustic transparency. The hydrogel samples were lowered into a water tank, where an ultrasound pulse was sent through the hydrogel and reflected by an acrylic wall back through the sample to be received by a transducer 70 mm away from the sample (Figure 7). Three different transducers were used and driven at their center frequencies of 1 MHz (WS-1P85, Ultran Group, State College, PA, USA), 3.5 MHz (Panametrics V381-SU, Olympus NDT Inc., Waltham, MA, USA) and 5 MHz (Panametrics V309, Olympus NDT Inc.) by a signal generator (33500 B, Keysight Technologies, Santa Rosa, CA, USA) at 10 Vpp, with 1 pulse every 10 ms. An oscilloscope (LeCroy Wavesurfer 44 Xs, Teledyne LeCroy, Chestnut Ridge, NY, USA) was used to log the reflected signal, and the acoustic attenuation a was measured by the loss of power in decibels compared to a reference measurement,

(1) $$a=\frac{1}{d}(20\log_{10}S_{ref}-20\log_{10}S),$$

with S and $S_{ref}$ being the Fourier transform of the reflected time-domain signal received as the ultrasound pulse went through the hydrogel and a reference sample, respectively. *d* is the length the sound travelled through the sample. The reference sample was a sample mold filled with water. Three to five samples were measured for each sample composition. The 2% (*w*/*v*) agarose with 10% (*v*/*v*) evaporated milk hydrogel gave the best compromise between acceptable optical transparency and increased acoustic attenuation. This hydrogel composition was used in all subsequent experiments, referred to as the agarose–milk hydrogel.

#### 5.3.2. Water Mass Fraction of Agarose–Milk Hydrogels

As an estimate of the porosity of the agarose–milk hydrogel, the mass fraction of water in the hydrogel was measured by comparing the wet weight and dry weight of the sample. 300 μL gel solution of the agarose–milk hydrogel in 1 mM PBS was pipetted onto a petri dish and immediately weighed to obtain the wet weight of the hydrogel. The hydrogel was allowed to set for 10 min at room temperature before being placed in a 40 °C heating cabinet. The samples were weighed daily for 7 days and subsequently weighed again after 7 days to ensure that the weight had stabilized, indicating complete drying of the sample. The water fraction $W_{H_{2}O}$ was estimated to be

(2) $$W_{H_{2}O}=\frac{m_{wet}-m_{dry}}{m_{wet}},$$

with $m_{wet}$ and $m_{dry}$ being the wet and dry weight of the hydrogel, respectively. Six samples were dried to estimate an average water fraction.

#### 5.3.3. Phase Contrast Imaging of Agarose–Milk Hydrogels

To investigate the distribution of fat globules in the agarose–milk hydrogels, samples of the hydrogel were imaged directly after gel formation using phase contrast microscopy (Eclipse TS100, Nikon, Tokyo, Japan) with a 40X objective (CFI S Plan Fluor ELWD ADM 40XC, Nikon) coupled to a camera (Digital Sight Fi1, Nikon). ImageJ [59] was used to measure diameters of the milk globules. 

#### 5.3.4. Hydraulic Conductivity of Agarose Hydrogels

**Figure 8:**
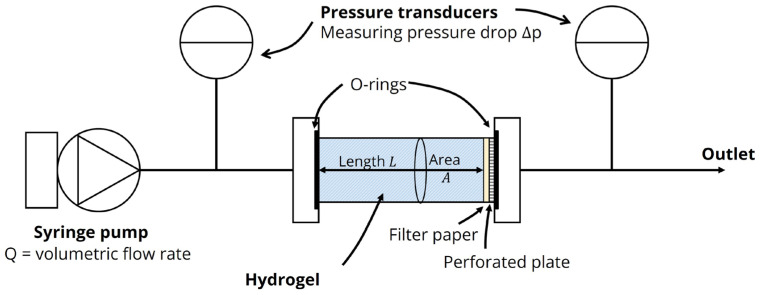
Measurement of hydraulic conductivity. Illustration of setup for measurement of hydrogel hydraulic conductivity. A syringe pump was used to drive fluid from a syringe through a hydrogel sample tube. The tube connections were sealed using O-rings, where the back end of the tube was mounted against a filter paper placed on top of a perforated plate to keep the hydrogel sample in place. Pressure transducers on both sides of the sample tube were used to monitor pressure drops across the sample.

The hydraulic conductivity is an important material characteristic for modeling flow in a porous medium and can be determined by relating the fluid flow to the pressure drop across the sample at steady state using the Darcy equation. A setup to measure hydraulic conductivity based on a similar setup by Lee et al. [39] was constructed (Figure 8). Hydrogel solutions were cast in tubes of 4 mm inner diameter and 10 mm length. The sample tube was inserted between two tube connections with luer-lock outlets and O-rings to ensure a leak-proof connection. The back end of the sample was supported by a perforated plate with a filter paper (Grade 4 Whatman, Sigma-Aldrich, St. Louis, MO, USA) on top to avoid sample movement during the measurements. Pressure transducers (Disposable BP Transducer, ADInstruments, Oxford, UK) were mounted on either side of the sample tube using luer-lock T-connections to monitor inlet and outlet pressure, logged to a computer through PowerLab 26 using the LabChart Software (ADInstruments). A syringe pump (PHD ULTRA, Harvard Apparatus, Holliston, MA, USA) was used to inject either DI water or 1 mM PBS through the sample from a 1000 μL gastight syringe (1000-series Gastight^®^ Hamilton, Sigma-Aldrich) at constant volumetric flow rates between 10 and 50 μL/h. To ensure near steady-state conditions for pressure measurements, the flow rate was kept constant for 1–2 h or 6 h for samples with and without evaporated milk, respectively. The hydraulic conductivity *K* could then be determined using the Darcy equation

(3) $$q=-K\frac{\Delta p}{L}\;,$$

where Δp is the pressure drop, ***q*** is the volumetric flux and *L* is the length of the sample. The hydraulic conductivity was measured in samples comprising 2% (*w*/*v*) agarose alone and with 10% (*v*/*v*) evaporated milk in 1 mM PBS. Samples of 2% (*w*/*v*) agarose in DI water were used as a reference case. Three samples were used in all cases.

### 5.4. Nanoparticle Imaging and FUS Treatment

#### 5.4.1. Confocal Imaging of Nanoparticles

**Figure 9:**
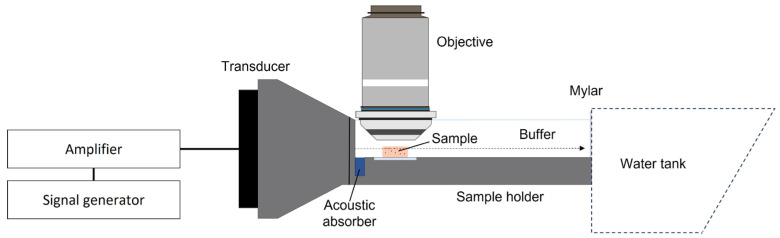
In situ imaging of nanoparticles in agarose–milk hydrogels during pulsed FUS exposure. Illustration of setup for confocal imaging of nanoparticles in hydrogels during FUS exposure. A 1 MHz transducer run by a signal generator through an amplifier was fitted into a water-filled cone. The cone was connected to a buffer-filled sample holder that placed the focus of the ultrasound field on the agarose–milk hydrogel, where the nanoparticles could be imaged in a plane parallel to the ultrasound field. The back wall of the sample holder was composed of Mylar that acted as an acoustic window to a large water tank (not drawn to scale) to allow for ultrasound beam propagation.

Nanoparticles in 1 mM PBS or in agarose–milk hydrogels were imaged using an upright confocal microscope (LSM 700, Zeiss, Oberkochen, Germany) with a 40X dip-in objective (W Plan-Apochromat 40X/1.0 DIC M27, Zeiss) of 2.5 mm working distance. The fluorescent particles were excited using a 555 nm laser with a 1.4 μm optical section and a 40 × 40 μm field of view, with fluorescence detection above 559 nm. For imaging of nanoparticles in agarose–milk hydrogels, the microscope stage was replaced with a custom ultrasound treatment setup for imaging during ultrasound exposure (Figure 9). Particles were imaged with a framerate of 4.5 frames per second for 5 min (1340 frames, 292 × 292 pixels). Nanoparticles suspended in buffer were imaged in a glass-bottom dish to compare freely diffusing particles to particles in the hydrogel. Videos of particles in buffer were recorded for 2 min at 2–3 different locations with a framerate of 20.5 frames per second (2600 frames, 252 × 252 pixels).

#### 5.4.2. Ultrasound Setup 

A custom-made setup for confocal imaging of polystyrene nanoparticles in the agarose–milk hydrogel during pulsed FUS exposure was constructed (Figure 9). A single-element focused transducer (center frequency: 1 MHz, focal depth: 73.5 mm, diameter 60 mm, Precision Acoustics, Dorchester, UK) was placed in a cone filled with degassed water and sealed with a thin plastic film. The transducer was driven by a signal generator (33500 B, Keysight Technologies) through a 50 dB power amplifier (2100 L from E&I Ltd., Rochester, NY, USA). A 3D-printed sample holder 9.5 cm long, 3.2 cm wide and 1.3 cm in height filled with 1 mM PBS was attached to the transducer cone. The sample holder positioned the agarose–milk hydrogel in the correct height relative to the focal point of the transducer, blocking approximately half of the opening of the transducer cone. An acoustic absorber (Aptflex F28, Precision Acoustics) was placed in front of the blocked section of the transducer cone to minimize ultrasound reflections. To ensure sufficient ultrasound beam propagation while minimizing standing waves, the back wall of the sample holder was replaced with an acoustically transparent Mylar sheet coupled to a larger water tank 19 cm in width, 25 cm in height and 34 cm in length, where the back wall of the tank was angled at 45°. 

Characterization of the transducer in the custom-made cone was performed using an Acoustic Intensity Measurement System (AIMS III) with an HGL-0200 hydrophone (Onda Corp., Sunnyvale, CA, USA). An acoustic absorber was placed in front of the cone during characterization in the same manner as in the treatment setup. The −3 dB beam width at the point of imaging was found to be 0.5 mm in diameter.

#### 5.4.3. Ultrasound Treatment

Agarose–milk hydrogels in 1 mM PBS with polystyrene nanoparticles were cast in wells formed by silicone isolators (Press-to-Seal, PSA on one side, Grace Bio-Labs, Sigma-Aldrich) 9 mm in width and 0.9 mm in depth. Following gel formation, the agarose–milk hydrogel was removed from the well, glued to an 18 × 18 mm cover slip and placed in a designated slit in the 3D-printed sample holder filled with 1 mM PBS. Nanoparticles in the hydrogel were imaged in a plane parallel to the ultrasound propagation direction at the center of the ultrasound focal point, 80 mm away from the transducer and 40–50 μm below the hydrogel surface. The nanoparticles were imaged for 5 min, the ultrasound was subsequently turned on and imaging continued for another 5 min during FUS exposure. The transducer was driven at 1 MHz, with a peak negative pressure of 1 MPa at the point of imaging. When using FUS and MBs for nanoparticle delivery, it is common to keep the DC and PRF low to allow for reperfusion of MBs into the tumor volume [14]. However, a range of DCs were tested here. The PRF was kept constant at 1 Hz, and the number of cycles were changed to obtain DCs of 0.1%, 1%, 10% and 20% (I_SPTA_ = 0.03, 0.34, 3.38 and 6.76 W/cm^2^) for the 100 nm particles. The ultrasound field was assumed to be uniform within the 40 × 40 μm imaging frame. To estimate whether the hydrogel itself could be displaced by FUS, large and immobile 500 nm particles were imaged before and during FUS treatment of the hydrogel with 10% and 20% DCs. The temperature in the buffer solution surrounding the gel was measured prior to and directly after FUS treatment using a thermocouple (2-channel thermometer TD242, type K, VWR International, Radnor, PA, USA). Three to five agarose–milk hydrogel samples were treated for each DC, with 20–60 nanoparticles in the field of view during imaging.

#### 5.4.4. Single-Particle Tracking and Estimation of Diffusion Coefficients

TrackPy [60] was used to localize and track particles between frames. Tracking parameters were tuned to filter out spurious particles and noise, tracking particles that were in the frame for more than 1.5–4 s for the fast-moving particles in buffer and 30 s for the slower moving particles in the agarose–milk hydrogel. Particle trajectories from replicate samples were combined into one file, giving roughly 100 or more individual particle tracks per experimental group. TrackPy was used to calculate the particle MSD and MD in both the x and y direction up to the first 100 time-steps and results were averaged to estimate the ensemble particle behavior. There were some uncertainties in the positioning of the sample holder relative to the imaging objective, and the imaging coordinate system had to be rotated slightly for each sample to ensure that the x direction (ultrasound propagation direction) coincided with the direction of particle displacement. The displacement velocity $v_{x}\;$of particles due to pulsed FUS was taken as the slope of the linear ensemble MD curve in the x direction. The overall drift velocity v of the ensemble was calculated as $v=\surd (v_{x}^{2}+v_{y}^{2})$, where $v_{y}$ is the slope of the MD curve in the y direction.

Curve fitting was conducted using GraphPad Prism (GraphPad Software version 10.0.2, San Diego, CA, USA), where the spread and number of particle tracks were used to estimate the uncertainty of the fitted parameters. To estimate the ensemble nanoparticle diffusion coefficient, the following expression was fitted to the measured ensemble *MSD*

(4) $$MSD=4Dt^{\alpha },$$

where D is the self-diffusion coefficient, t is the time-step or lag time and α is a coefficient describing the power law behavior of self-diffusion. In the case of pure Brownian motion, α=1, while α<1 is indicative of anomalous subdiffusion [61]. For nanoparticles in the hydrogel samples, the overall drift was subtracted from the MSD curve prior to curve fitting [28,29]. For the particles not exposed to ultrasound, curve fitting was conducted by varying both D and α freely. The α of nanoparticles in the FUS-exposed hydrogel samples was kept constant and equal to the no FUS case, with D being the only free parameter in the curve fit. For particles in buffer, Equation (4) was only fitted to the first 25% of the ensemble MSD curve to avoid artefacts arising due to low statistics at longer time-steps.

To test for any significant increase in the diffusion coefficient with application of FUS compared to the no FUS case, a two-sampled one-sided Z-test was performed using the curve fitted parameters and the estimated error in the curve fit. A *p*-value below 0.05 was considered statistically significant.

### 5.5. Volume Averaged Model of Acoustic Streaming

The measured nanoparticle displacement velocities were compared to a previously developed model of acoustic streaming [35]. In our previous work, we derived equations describing acoustic streaming in soft porous media driven by FUS, and the reader is referred to this work for the full derivation of equations. Briefly, starting from the well-established perturbation approach [18,62], applying the perturbation to the conservation of mass and momentum balance and performing a volume averaging procedure [63], the following equation for acoustic streaming in the hydrogel was derived

(5) $$q=-K\left.\left(\left.\nabla \langle p_{2}\rangle \right.-\langle F_{2}\rangle \right.\right).$$

Here, q is the volumetric flux and K is the hydraulic conductivity of the porous medium set to the experimentally measured value of 0.41⋅10^−12^ m^2^/Pa⋅s (Figure 2B). Further, $\left.\langle p_{2}\rangle \right.$ and $\langle F_{2}\rangle$ denote temporal and spatially averaged second-order pressure and ARF, respectively. In instances where the focal point is long in the axial direction compared to its width, the second-order pressure can be neglected [35]. This is the case for the transducer used in this experimental setup, which gives

(6) $$q\approx K\langle F_{2}\rangle .$$

Using the paraxial (or parabolic) approximation, the following relation between the ARF and the intensity I is obtained

(7) $$\langle F_{2}\rangle =\frac{2aI}{c_{0}},$$

where the acoustic attenuation coefficient a was set to the measured value of 0.14 dB/cm (Table 1) and $c_{0}$ is the speed of sound set to 1480 m/s. The intensity can be approximated by plane-wave relations to

(8) I=P122ρ0c0x^,

where $P_{1}$ is the amplitude of the acoustic pressure which was set to be 1 MPa corresponding to the experimental setup measurement of the peak negative pressure 80 mm away from the transducer. x^ denotes the unit vector in the x direction, corresponding to the ultrasound propagation direction. Combining Equations (6)–(8) gives an estimate of the acoustic streaming velocity

(9) v=KaP12ϕρ0c02x^ ,

where $\rho_{0}$ is set to 1000 kg/m^3^ and ϕ is the porosity set equal to the measured mass fraction of water at 0.953. Equation (9) was multiplied by the DC to estimate the acoustic streaming with pulsed FUS. 

## Figures and Tables

**Table 1:** Measured acoustic attenuation of agarose hydrogels with added bovine serum albumin (BSA) or evaporated milk at 1, 3.5 and 5 MHz.

Agarose Concentration	Additive	Additive Concentration	Acoustic Attenuation (dB/cm)		
			1 MHz	3.5 MHz	5 MHz
2 % *w*/*v* (*n* = 3)	-	-	0.04 ± 0.02	0.10 ± 0.03	0.35 ± 0.07
	BSA	10% *w*/*v*	0.11 ± 0.03	0.32 ± 0.02	0.44 ± 0.05
		20% *w*/*v*	0.10 ± 0.03	0.52± 0.03	0.81 ± 0.12
	Evaporated milk	5% *v*/*v*	0.04 ± 0.01	0.16 ± 0.06	0.48 ± 0.12
		10% *v*/*v*	0.14 ± 0.02	0.41 ± 0.02	0.51 ± 0.09
5 % *w*/*v* (*n* = 5)	-	-	0.02 ± 0.01	0.49 ± 0.03	1.01 ± 0.09
	Evaporated milk	10% *v*/*v*	0.11 ± 0.04	0.54 ± 0.29	1.37 ± 0.12
		20% *v*/*v*	0.18 ± 0.04	0.82 ± 0.24	1.73 ± 0.07

## Data Availability

Data available upon request.

## Appendix A. Effect of Acoustic Streaming above the Hydrogel

The images used for the nanoparticle tracking were taken 40–50 μm below the gel surface. As the gel was submerged in buffer, there will be a homogeneous liquid acoustic streaming above the gel surface which will be some orders of magnitude larger than the acoustic streaming in the gel. The effect of viscous coupling to flow in buffer can be estimated by calculating the flow near the top of the gel using the Darcy–Brinkman equation [64,65]

(A1) $$-\nabla p+\eta \nabla^{2}v-\frac{\eta }{\kappa }v=0,$$

where p is the pressure, η is the dynamic viscosity, κ is the permeability and v is the fluid velocity. Let *x* be the ultrasound propagation direction, *y* be the direction of elevation and assume there is only flow in the x direction that varies with *y*. Equation (A1) can then be rewritten as

(A2) $$\frac{d^{2}v_{x}}{dy^{2}}=\frac{1}{\eta }\frac{dp}{dx}+\frac{v_{x}}{\kappa }.$$

The solution to this equation is

(A3) $$v_{x}\left(y\right)=\left.\left\{v_{0}-v_{\infty }\right.\right\}\exp(y/\sqrt{\kappa })+v_{\infty },$$

where $v_{0}$ is the flow velocity at the gel–buffer interface at *y* = 0, and

(A4) $$v_{\infty }=-\frac{\kappa }{\eta }\frac{dp}{dx}.$$

This equation can be solved for the distance Δy down into the gel for the effect of the buffer flow to decrease by one order of magnitude,

(A5) $$\frac{v\left(y-\Delta y\right)-v_{\infty }\;}{v\left(y\right)-v_{\infty }}=0.1.$$

Solving for Δy gives

(A6) $$\Delta y=-\sqrt{\kappa }\log\left(0.1\right).$$

With κ=Kη from our experimental setup, and taking η = 10^−3^ Pa⋅s (for water at room temperature), then Δy = 47 nm. The images from the experiment were taken 40 μm into the gel, where the effect of flow in the buffer should have vanished completely.
